# Application of Mac-2 binding protein glycosylation isomer as a non-invasive biomarker for probing liver disease

**DOI:** 10.1038/s41598-022-10744-5

**Published:** 2022-04-26

**Authors:** Kawin Tangvoraphonkchai, Tanita Suttichaimongkol, Churairat Kularbkaew, Prakasit Sangaimwibool, Wattana Sukeepaisarnjaroen

**Affiliations:** 1 Division of Gastroenterology, Department of Medicine, Faculty of Medicine, Khon Kaen University, Khon Kaen, Thailand; 2 Department of Pathology, Faculty of Medicine, Khon Kaen University, Khon Kaen, Thailand

**Keywords:** Hepatology, Biochemistry

## Abstract

Liver disease remains a major critical challenge in Thailand due to viral hepatitis. Clinical management requires close monitoring of liver fibrosis severity. Non-invasive testing is an attractive method for probing of disease progression. Mac-2 binding protein glycosylation isomer (M2BPGi) is a novel serum marker for fibrosis staging. The current study evaluates the marker among healthy donors and hepatitis C (HCV) patients. 100 HCV subjects were evaluated by liver biopsy. These patients had varying fibrosis severity based on METAVIR scores. Healthy donors were confirmed based on normal liver functions tests. Comparisons of M2BPGi levels among different study groups were performed and the effectiveness was evaluated using receiver operating characteristics (ROC) curves. Using liver biopsy as the reference standard, median M2BPGi levels in HCV cases were 0.74, 1.38 and 2.88 COI for F0-1, F2 and > F3 cases respectively. In healthy donors, the baseline values ranged 0.1–0.24 COI and statistically lower than liver disease cases profiled using M2BPGi. ROC analysis demonstrated superior results for M2BPGi levels among diseased populations and healthy controls. AUROC was determined at 0.983. Comparing with other non-invasive tests, M2BPGi showed a positive linear trend that indicated a strong match to existing methodologies. M2BPGi addresses a critical need in the management of liver disease by providing straightforward means to probe fibrosis severity. In this study, we found significant differences between hepatitis C and healthy subjects and established the background level in healthy donors.

## Introduction

Chronic liver disease progresses to liver fibrosis, cirrhosis, and finally hepatocellular carcinoma. Every year, chronic liver disease causes 2 million deaths globally, of which one million are due to cirrhosis and one million are due to viral hepatitis and hepatocellular carcinoma^1^ while liver cancer is the sixth most commonly diagnosed cancer and the fourth leading cause of cancer deaths (8.2% of cancer deaths) worldwide in 2018^2^. In Thailand, there are approximately 600,000 hepatitis C (HCV) patients^3^ that are a cause of concern for complications and cancer. In addition, the prevalence of non-alcoholic fatty liver disease (NAFLD) is increasing in Asia due to increasing prevalence of obesity. This will inevitably lead to an increase in the number of patients with cirrhosis and cancer^4^. Hence, the economic burden of liver diseases such as HCV^5^ and non-alcoholic steatohepatitis (NASH)^6^ are high in Thailand.

Early diagnosis and treatment are of paramount importance in stopping the progression of liver disease to cirrhosis and liver cancer. Chronic HCV can be treated with direct-acting antivirals (DAAs) such as sofosbuvir/velpatasvir and glecaprevir/pibrentasvir. Current guidelines recommend DAAs for chronic HCV treatment regardless of fibrosis stage. The control of viral loads is effective with such treatments, but liver diseases have manifested in these chronic patients that need to be addressed. Hence, liver fibrosis staging is important in the management of patients with chronic liver disease. At present, the gold standard for liver fibrosis staging is a liver biopsy. However, liver biopsy has its limitations, such as high cost, sampling error, inter-observer variations, and the risk of complications^7^. It is also not feasible to perform liver biopsies repeatedly for monitoring of disease progression. Other methods for liver fibrosis staging include transient elastography^8^, acoustic radiation force impulse^9^, serum biomarkers such as hyaluronic acid and lincRNA-p21^10^. These methods require expensive equipment, are time-consuming, and have low specificity and sensitivity. Therefore, they may not be useful in resource-poor settings where liver disease burden is high. It is also difficult to integrate into existing healthcare screening practices since patients will need additional time on site to perform such tests.

Mac-2 binding protein glycosylation isomer (M2BPGi) is a biomarker that was identified in patients with liver fibrosis^11^. In addition, studies have shown that M2BPGi is correlated with liver fibrosis stages^12^, is a predictor of lenvatinib tolerability and response in hepatocellular carcinoma^13^, regression of liver fibrosis after treatment in HCV^14^, is associated with advanced liver fibrosis in diabetes^15^, is correlated with liver stiffness^16^, and is a biomarker for survival after HCV eradication^17^, demonstrating the usefulness of this novel biomarker. M2BPGi is also a reimbursable clinical test in Japan but limited data is available in other Asia Pacific countries as highlighted in the 2016 Asian-Pacific Association for the Study of the Liver (APASL) consensus guidelines. Further studies in comparison to other non-invasive modalities were also recommended by the panel. In this study, we aim to establish clear clinical relevance of M2BPGi in the Thai population. Building upon prior M2BPGi research work, we established and examined M2BPGi levels within HCV patients in comparison to healthy people in Thailand. These determine the usefulness of the biomarker in the diagnosis of liver disease. In addition, the comparison results with other non-invasive alternatives showed interesting trends that were not previously highlighted.

## Materials and methods

### Study design and patient demographics

**Figure 1:**
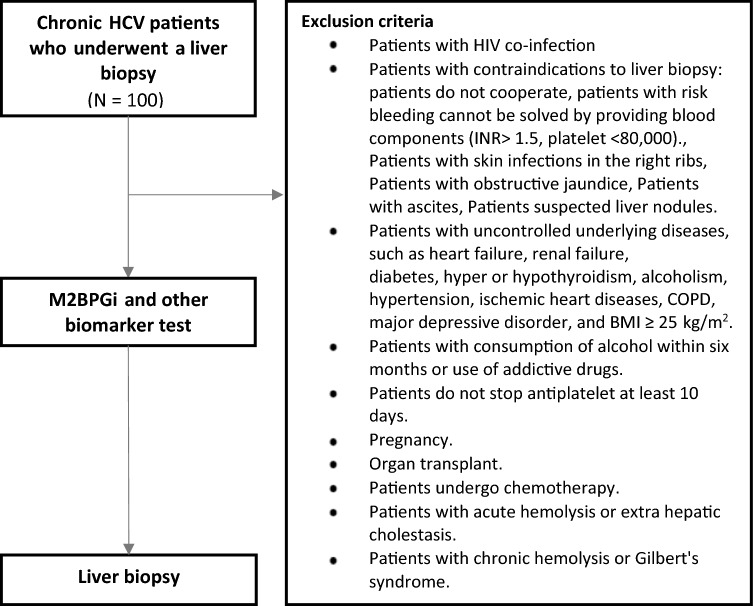
The study design to investigate the usefulness of M2BPGi in Thai liver disease patients.

100 subjects with HCV were recruited for this study between June 2017 and December 2019. These patients have mixed liver fibrosis stages based on METAVIR scores. This study is a prospective, single center diagnostic study with approval by the Khon Kaen University Ethics committee for Human Research based on the Declaration of Helsinki and the ICH Good Clinical Practice Guidelines. (HE591548). Informed consent was obtained from all study subjects. Liver tissues were used as the comparator as this method is the reference gold standard. Other commonly used non-invasive liver disease testing modalities were concurrently collected from these groups of patients. Inclusion criteria for HCV patients were for treatment naïve cases and of mono-infection. The list of exclusion criteria are shown in Fig. 1. 63 Healthy volunteers were used as controls in this study and liver function in these volunteers was verified by hepatic function tests (Table 1), serology (HBsAg, Anti-HBs, Anti-HBc, Anti-HCV, Anti-HIV), and ultrasound.

**Table 1:** Baseline characteristics of the 100 HCV patients.

Characteristics	Values
Male:Female	67:33
Age (years)	51.0 (8.3)
**Underlying disease**	
None	77 (77)
Diabetes mellitus	11 (11)
Hypertension	12 (12)
Hemoglobin (g/dl)	13.8 (1.4)
Platelet count (10^3^/mm^3^)	192.5 (56.5)
Creatinine (mg/dl)	0.9 (0.2)
Albumin (g/dl)	4.4 (0.4)
Total bilirubin (mg/dl)	0.7 (0.3)
ALT (U/L)	73.2 (54.8)
AST (U/L)	63.6 (43.3)
ALP (U/L)	90.0 (36.8)
INR	1.0 (0.1)
HCV Viral load (IU/ml)	4,032,315 (5,919,394)
**HCV genotypes**	
1A	15 (15)
1B	17 (17)
3	40 (40)
6	28 (28)
**Fibrosis score (METAVIR)**	
F0	7 (7)
F1	28 (28)
F2	33 (33)
F3	17 (17)
F4	15 (15)
**Liver assessment**	
APRI	1.18 (0.95)
FIB4	2.27 (1.37)
FibroIndex	1.76 (0.57)
Fibroscan (kPA)	13.39 (11.85)
Fibrotest	0.53 (0.28)
M2BPGi (COI)	1.97 (2.07)

Data are expressed as mean (standard deviation) or number (%).

ALT = alanine aminotransferase, AST = aspartate aminotransferase, ALP = alkaline phosphatase, INR = international normalized ratio.

### M2BGPi biomarker measurements

Serum M2BPGi levels were measured on the HISCL 5000 automated immunoassay analyzer (Sysmex Corporation, Japan). 10 μL of sample were used and M2BPGi levels were measured by a sandwich immunoassay. Each reaction took 17 min and M2BPGi was expressed as the cut-off index (COI) and calibrated using the manufacturer’s calibrators. Residual samples from routine blood sampling were used and remaining serum samples stored at −20 °C prior to processing on the instrument. Samples were batched processed at a rate of 200 tests per hour on the HISCL 5000. From manufacturer’s claim, results above 1.0 COI indicate a risk of liver fibrosis.

### Fibroscan of study subjects

All patients had liver stiffness measured by FibroScan^®^ 502 touch (Echosens, France) using the M or XL probe according to patients’ body mass index (XL probe for body mass index ≥ 25). This was conducted by experienced nurses (who had conducted more than 100 cases each) on the same day liver biopsy was performed on study subjects (NPO of more than 8 h on day of procedure). The report showed E score (liver stiffness) to interpret liver fibrosis stage.

### Liver disease biomarker tests

20 cc of blood specimens were collected from each test subject including healthy controls. For liver biopsy requirements and consistency, subjects were told to abstain from food 8 h before (NPO > 8 h). Blood samples were used in various laboratory assays to evaluate the comparative indirect liver fibrosis markers such as Fibrotest (LiverFact, Fibronostics Singapore) that defined significant liver fibrosis (F2 METAVIR score) with scores > 0.48, APRI (AST to platelet ratio index), Forns index and FIB-4 (The Fibrosis-4 Index). Blood tests were also necessary for basic liver function test analysis and preparation for liver biopsy.

### Liver biopsy

Liver biopsy was performed on patients who did not have contraindication for the procedure. Patients were laid on their backs and given NSS intravenously at 120 ml/hr. Ultrasound was done to check for liver parenchyma and to mark the area for liver biopsy. These patients were given midazolam 2.5 mg and pethidine 25 mg intravenously before liver biopsy. Percutaneous liver biopsy was performed with a 1.6 mm diameter Trucut needle with a length of at least 2.5 cm. All liver biopsies were at least 8 portal triads and a length ≥ 2.5 cm. All specimens were fixed in formalin, embedded in paraffin, cut, and stained with hematoxylin and eosin. All liver histology interpretations were performed by two pathologists independently (K. Churairat and S. Prakasit) who had extensive experience in reading biopsied liver samples. Disagreements between pathologists were resolved through discussion and mutual decision about results. Reports were based on the METAVIR scoring system, which specifies a fibrosis score from 0 to 4.

### Statistical analysis

All variables were expressed as mean ± SD unless otherwise defined. The Kruskal–Wallis one-way analysis of variance (ANOVA) was used to compare multiple independent groups as we cannot ascertain normality from our dataset. The Mann–Whitney test was used to compare if there existed a difference in the dependent variable for two independent groups. *P* values < 0.05 were considered statistically significant in these investigations. Comparative analysis among healthy controls and diseased patients was computed using Receiver operating characteristics (ROC) curves and plotted to determine the area under ROC (AUROC) curves. These analyses were conducted to compare HCV patients with varying degree of liver fibrosis as well as with healthy control data. Spearman’s rank-order correlations were used to determine the correlations between M2BPGi measurements and other non-invasive liver fibrosis markers. Statistical analyses were performed with STATA^®^ 10.1 software (StataCorp, USA).

## Results

### Patient background data

Table 1 presents the patient subject demographics. A total of 100 patients were enrolled with none of them were excluded. Among them, 67 were male (67%) and 33 female (33%), and were all HCV positive. The average patient age was 51.0 ± 8.3 years. Genotyping were performed and shown to be predominantly genotypes 3 and 6 (genotype 1a, 1b, 3 and 6 in 15 (15%), 17 (17%), 40 (40%) and 28 (28%) patients, respectively). Figure 1 shows the study design to investigate the usefulness of M2BPGi in Thai liver disease patients. Patients were selection for mono-infection cases only and excluded if they had HIV co-infections, unable to perform liver biopsy, had uncontrolled underlying conditions, pregnant cases or patients on other therapies. Patients were treatment naive at recruitment and mean viral load among these patients were 4,032,315 IU/mL. We performed liver biopsies in all 100 patients, and fibrosis stages were F0 (7, 7%), F1 (28, 28%), F2 (33, 33%), F3 (17, 17%) and F4 (15, 15%). An additional 63 healthy controls were considered, all of which had LFTs within normal reference intervals and normal ultrasound upper abdomen (Table 2).

**Table 2:** Baseline characteristics of the 63 normal volunteers.

Characteristics	Values
Male:Female	17:46
Age (years)	48.11 (11.06)
Body Mass Index (kg/m^2^)	21.62 (2.07)
Hemoglobin (g/dl)	12.67 (1.44)
Platelet count (10^3^/mm^3^)	282.97 (93.87)
Creatinine (mg/dl)	0.8 (0.16)
Cholesterol (mg/dl)	175.94 (19.72)
Albumin (g/dl)	4.37 (0.21)
Total bilirubin (mg/dl)	0.54 (0.12)
ALT (U/L)	17.79 (5.48)
AST (U/L)	20.67 (4.3)
ALP (U/L)	64.54 (15.81)
INR	0.96 (0.34)

Data are expressed as mean (standard deviation) or number (%).

ALT = alanine aminotransferase, AST = aspartate aminotransferase, ALP = alkaline phosphatase, INR = international normalized ratio.

### Low baseline M2BPGi levels among healthy subjects

**Figure 2:**
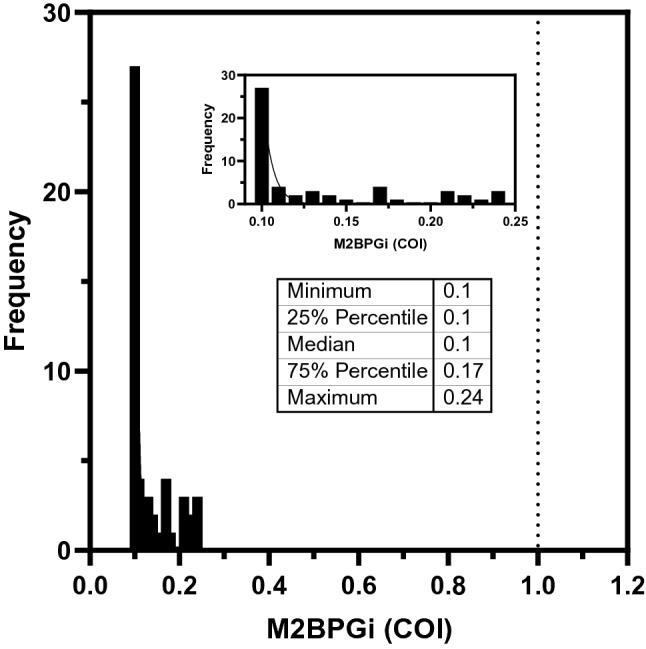
M2BPGi levels among healthy volunteers which is significantly below the cutoff (1.0 COI) recommended by the manufacturer for positive results.

Baseline M2BPGi levels were established through healthy donor measurements. Figure 2 shows the spread of results among all 63 datapoints. Median M2BPGi levels were 0.1 COI and the range was observed to be between 0.1 to 0.24 COI, well below the recommended cutoff of 1.0 COI that indicates risk of liver disease. Further examination of the dataset showed that results were skewed towards the left, with majority of datapoints below 0.25 COI in healthy Thai subjects. This baseline dataset allows a direct comparison to diseased patients to establish suitable cutoffs for disease assessment.

### Positive correlation with other liver fibrosis markers

**Figure 3:**
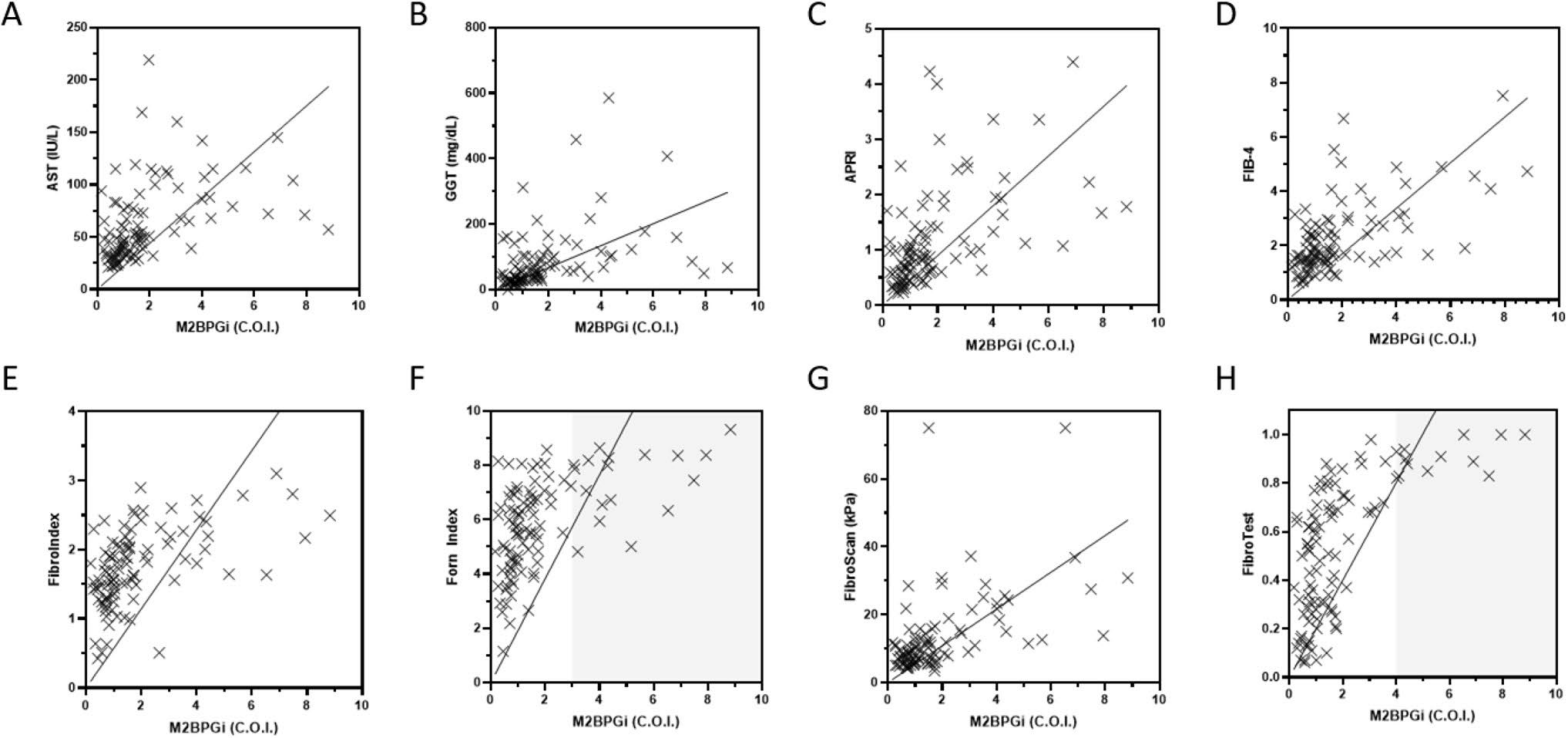
Comparison of M2BPGi levels among other non-invasive measures of liver fibrosis assessment. (**A**) Comparison with AST, (**B**) GGT, (**C**) APRI, (**D**) FIB-4, (**E**) FibroIndex, (**F**) Forns Index, (**G**) Fibroscan and (**H**) Fibrotest. Results showed positive correlations using a Spearman’s rank order correlation.

Raw data values of M2BPGi were correlated to commonly used non-invasive techniques for liver disease assessment. This is presented in Fig. 3. These associations included comparisons with AST (Figure A); GGT (Figure B); APRI (Figure C); FIB-4 (Figure D); Fibroindex (Figure E); Forns Index (Figure F); Fibroscan (Figure G); and Fibrotest (Figure H). Spearman’s rho among these parameters ranged from 0.564 to 0.686, which indicated positive correlations of M2BPGi measurements to each of the liver disease assessment methods. Mean M2BPGi levels obtained was 1.98 ± 2.06 COI that spans from patients from F0 (no fibrosis) to F4 (cirrhosis) conditions. In three of such comparisons (Fibroindex, Forns index and Fibrotest), we observed that the comparator assays plateaued approximately after M2BPGi levels at 3.0 COI. The results highlight potential clinical utility to utilize M2BPGi measurements in staging liver fibrosis cases.

### Estimation of diagnostic accuracy in chronic hepatitis C cases

**Figure 4:**
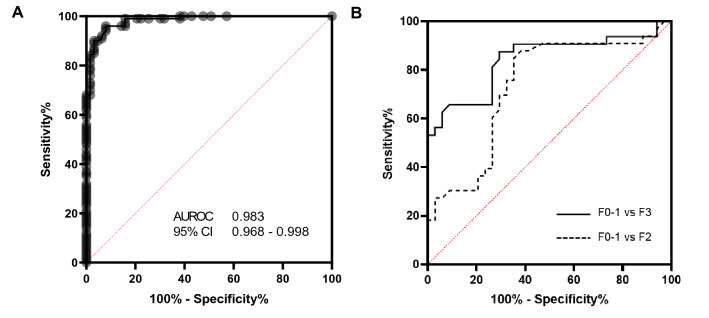
Receiver operating characteristics (ROC) curves analysis comparing (**A**) HCV study cohort (AUROC = 0.983) with controls and (**B**) early liver fibrosis cases with moderate (F2), or severe (F3) and cirrhotic (F4) cases. AUROC of 0.73 and 0.84 obtained for F0-1 against F2 and ≥ F3 cases respectively.

**Figure 5:**
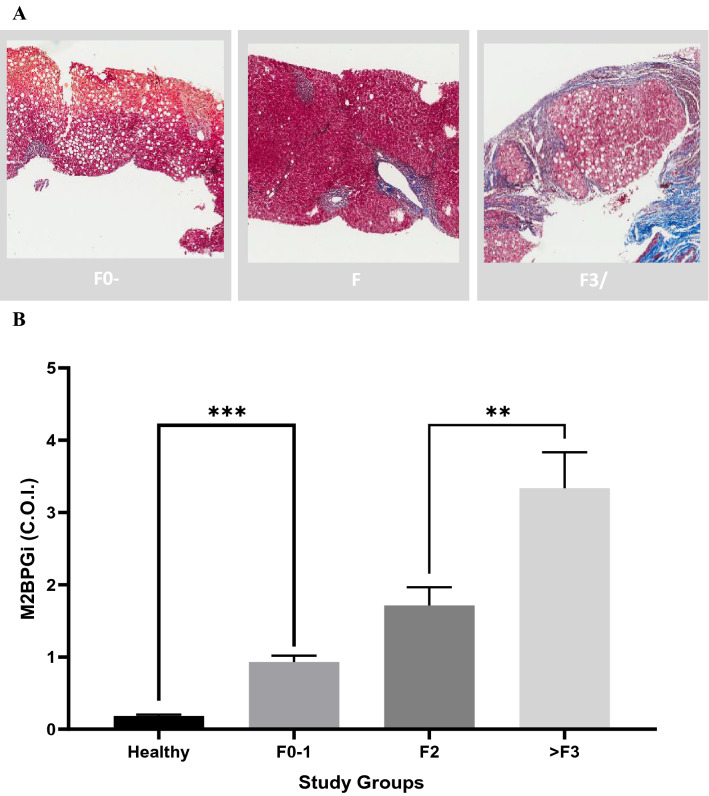
Comparison of M2BPGi levels with Metavir scoring from histopathological evaluation in liver biopsy of patients. (**A**) Representative histological images of F0-1 (no fibrosis), F2 (enlargement of the portal tract with rare septa formation) and F3/4 case (cirrhosis). (**B**) Corresponding average levels in each study group. SEM plotted. Kruskal Wallis one-way ANOVA shows significant differences among study groups. A Mann–Whitney test between patients with significant liver fibrosis and severe fibrosis/cirrhosis showed statistical significance (*p* = 0.005) and between healthy and early disease (*p* < 0.0001).

We attempted to assess the diagnostic accuracy of M2BPGi in comparisons with healthy subjects. A ROC analysis was performed as shown in Fig. 4A. M2BPGi levels were vastly discriminatory against chronic HCV cases with AUROC of 0.983 (95% CI of 0.968 to 0.998). Mean values of AST and ALT (Table 1), which are part of basic liver function assessments are significantly elevated (*p* < 0.001) in the patient cohort compared with the upper limit of normal (ULN). Further discriminatory analyses were conducted among HCV groups of patients of varying degree of liver fibrosis (Fig. 4B). We observe AUROC of 0.73 and 0.84 for patient groups F0-1 against F2 and ≥ F3 cases respectively. These results highlight the excellent sensitivity and specificity of such tests in differentiating various degree of liver disease. In addressing suitable liver fibrosis cutoffs using M2BPGi and associating with histopathology results, we categorized HCV patients into different fibrosis severities as shown in Fig. 5 similar to what was conducted using the ROC analysis. Liver biopsy data provided definitive assessment of patient’s condition using current gold standard methodology. Median M2BPGi cutoffs of F0-1, F2 and > F3 cases were 0.74 COI, 1.38 COI and 2.88 COI respectively. A Kruskal Wallis test showed statistical significance between different study groups (*p* < 0.0001). Further assessments using non-parametric Mann–Whitney analyses (one tail) indicated statistical significance for higher values of > F3 cases against F2 (*p* < 0.01) and F0-1 against healthy controls (*p* < 0.01). This provided strong evidence to segregate each cohort using M2BPGi measurements, especially addressing early disease (F0-1 cases) and detecting patients with severe fibrosis or cirrhosis (> F3 cases).

## Discussion

Non-invasive tests for measuring liver fibrosis stage are attracting more and more attention. This technology is particularly useful in detecting liver fibrosis in remote regions with high incidence of HCV, such as Thailand. The HCV seroprevalence is 0.9%, which is 760,000–1,475,000 anti-HCV positive patients^5^. In addition, more than half the patients (52.2 to 62.5%) had advanced liver fibrosis (F3 and F4) in areas with high (12%) and average (2%) HCV viremia prevalence^5^. This shows that many patients in Thailand are not treated until advanced liver fibrosis, which results in greater morbidity and mortality.

A bottleneck in the treatment of such patients is identification of HCV patients and determining their liver fibrosis stages as most cases occur in rural regions of Thailand. The advent of DAAs has tremendously changed the management of chronic HCV and in the foreseeable future, liver fibrosis will be better controlled in this group. Reaching this group of patients is crucial to drive complete HCV elimination and integrative care for liver disease will be beneficial to prevent progression into advance stages. M2BPGi is attractive from this point of view as blood sampling would have been collected for serology testing of anti HCV, and can be subsequently used in positive cases for fibrosis assessment. Our results demonstrated clear distinct differences among HCV patients with varying degree of liver fibrosis manifestations and healthy subjects. This clear distinction allows early warning flags for risk that can be addressed by referral these patients to hepatologists. In addition, M2BPGi levels are correlated with other markers of liver fibrosis and ultrasound. This is consistent with the results of previous studies in Vietnam^18^, Thailand^19^, Japan^20^, and Egypt^21^. However, these studies used surrogate markers of liver fibrosis instead of liver biopsy, which is the gold standard. The present study is the first one to show a correlation between M2BPGi levels and liver fibrosis as assessed by liver biopsy outside of Japan, which increases its reliability and demonstrates the utility of using M2BPGi as a surrogate marker for liver fibrosis.

Since 2012, the National Health Security Office of Thailand has included PEGylated-interferon (PEG-IFN) based therapy for HCV into the Universal Health Coverage program. However, this policy has constraints such as the stringent screening eligibility criteria that consist of requirements related to severity and progression of liver damage. Therefore, the use of M2BPGi level to determine liver damage could circumvent this constraint and allow more HCV patients to be treated. The decreasing cost of DAAs may help increase treatment availability to more patients in Thailand. In fact, there is a need for a national plan for identification and eradication of HCV, as evidenced by a report by the Coalition to Eradicate Viral Hepatitis in Asia Pacific in 2015. In that whitepaper, a lack of epidemiological data is cited as one of the barriers for elimination of HCV^3^. The promotion of M2BPGi as a liver fibrosis method can help in the identification of liver fibrosis patients and aid in HCV elimination. Our results indicated possible uses in detecting early disease as significantly different M2BPGi levels were observed comparing early liver fibrosis cases with healthy controls. Identifying early cases link patients to care immediately that will greatly improve clinical outcomes. M2BPGi assay provides a straightforward means to profile hepatitis patients using the same blood sample without further adding on to patient’s burden. In identifying severe or cirrhotic cases, the distinctions are clear with median M2BPGi levels in > F3 cases to more than 2 times that of F2 cases. As we observed in the comparisons for Fibroindex, Forns Index and Fibrotest, these tests saturated for cases with high M2BPGi levels. The clear advantage for M2BPGi is the larger dynamic range to profile late-stage patients. This potentially addresses disease monitoring cases that aims to control the progression of liver disease.

In fact, studies found that liver fibrosis regression occurs when DAAs are used for HCV treatment^14,19,21^ and M2BPGi is a marker for patient survival after treatment with DAAs^17^. One paper even found that M2BPGi has a better performance than APRI in monitoring liver fibrosis^19^ while another paper found that M2BPGi shows better performance than other markers in assessing liver fibrosis in HCV patients^22^. Most studies on M2BPGi levels and HCV were on patients treated with DAAs. However, a study from Japan showed that M2BPGi level was equally useful when HCV patients were treated with IFN-based therapies^23^, which are mostly used in Thailand.

One limitation of this study is that it is a single-center study involving two cohorts (HCV and healthy subjects) and a larger, multicenter study is required for verification of the results as Thailand is a large country with different regions. In addition, the subjects in this study were Thai and the results may not be extrapolated to our ethnicities. In fact, most of the studies performed on M2BPGi were in Asian populations and more studies should be conducted in other races to determine the optimal cutoff indexes for different liver diseases in these populations. Another limitation is our assessment of healthy controls which are rudimentary consisting of liver function tests and ultrasound. More stringent criteria may be applied in other countries, but our current data shows M2BPGi levels are significantly lower than HCV patients. This cutoff as demonstrate in the ROC analysis show good sensitivity and specificity to rapidly identify possible liver fibrosis cases.

## Conclusion

Our study results showed that M2BPGi levels can be used to differentiate between HCV patients and healthy subjects and determine fibrosis stage in HCV patients. The present study is the first to show a correlation between M2BPGi level and liver fibrosis stage as assessed by biopsy other than the limited dataset from Japan. This technique can be used to track regression of liver fibrosis during HCV treatment, which is useful in resource-poor settings where prevalence of HCV and advanced liver fibrosis are high.
